# A transferable quantum mechanical energy model for intermolecular interactions using a single empirical parameter

**DOI:** 10.1107/S2052252523008941

**Published:** 2023-10-31

**Authors:** Peter R. Spackman, Mark A. Spackman, Julian D. Gale

**Affiliations:** a School of Molecular and Life Sciences, Curtin University, Perth, Western Australia 6845, Australia; b School of Molecular Sciences, University of Western Australia, Perth, Western Australia 6009, Australia; ESRF, France

**Keywords:** computational modelling, lattice energy, molecular crystals, intermolecular interactions

## Abstract

The calculation of intermolecular interactions in molecular crystals using model energies provides a unified route to understanding the complex interplay of driving forces in crystallization, elastic properties and more. Presented here is a new single-parameter interaction energy model (CE-1p), extending the previous *CrystalExplorer* energy model and calibrated using density functional theory (DFT) calculations at the ωB97M-V/def2-QZVP level over 1157 intermolecular interactions from 147 crystal structures. The new model incorporates an improved treatment of dispersion interactions and polarizabilities using the exchange-hole dipole model (XDM), along with the use of effective core potentials (ECPs), facilitating application to molecules containing elements across the periodic table (from H to Rn). This new model is validated against high-level reference data with outstanding performance, comparable to state-of-the-art DFT methods for molecular crystal lattice energies over the X23 set (mean absolute deviation 3.6 kJ mol^−1^) and for intermolecular interactions in the S66x8 benchmark set (root mean-square deviation 3.3 kJ mol^−1^). The performance of this model is further examined compared to the GFN2-xTB tight-binding model, providing recommendations for the evaluation of intermolecular interactions in molecular crystal systems.

## Abstract 2

A new interaction energy model for molecular dimers, CE-1p, accurately calculates intermolecular interactions in molecular crystals. Improved treatment of dispersion and polarization helps it outperform existing models with accuracy comparable to advanced DFT methods with only a single fitted parameter.

## 1. Introduction

A detailed quantitative description of intermolecular interactions – at low computational cost – is essential to the modelling of molecular crystals and many other chemical problems. This includes rationalization of crystal packing, the factors driving crystal growth and dissolution, mechanical properties of crystals and more. The study of these inter­actions is well established, but the prediction and understanding of the relative energetic contributions and terms driving these interactions is as important as ever. Moreover, there is an ever-expanding taxonomy of intermolecular interactions within the study of molecular crystals (and more generally), which now include not only terms such as hydrogen bonding and π–π stacking but also halogen, chalcogen, pnictogen and even aerogen bonding. Thus, accessible and accurate methods to compute both intermolecular interaction energies and their substituent factors are extremely useful, not only for their predictive power but also for their capacity to provide a unified view of intermolecular interactions as not simply a collection of categorized interaction types or intermolecular ‘bonds’, but in terms of the fundamental underlying physical forces and concepts.

Energetic approaches to understanding and rationalizing intermolecular interactions can be broken down broadly into two categories, composition and decomposition. The former encompasses models that build up the total interaction energy from prediction of separate components, and the latter encompasses those that start from a total energy and break it down into components after the fact. There are a myriad approaches to this end, with some components (*e.g.* Coulomb or electrostatic interactions) being more universally present than others. Further, the boundary between these two approaches is often unclear – some terms may be naturally more separable than others due to their mathematical/physical derivation and, likewise, it is always possible to produce decompositions that may be more artificial than meaningful. In either case, though, the motivation is not only to predict the total energy of a given system, but also to understand the components which lead to this energy and therefore, in the context of a molecular crystal, rationalize why a particular interaction may be present (or not) in the experimentally observed structure.

For molecular crystalline solids, a natural composition (or decomposition) approach is to consider the total energy as a sum over all pairwise dimer energies within a given range. Calculations of conventional quantum mechanical (QM) dimer interaction energies involve three full self-consistent field (SCF) calculations, where the interaction energy of the system is computed as the difference between the total energy of the dimer, *AB*, and the sum of the total energies of the monomers, *A* and *B*, *i.e.* *E*_int_ = *E*_*AB*_ − (*E*_*A*_ + *E*_*B*_). In the absence of a complete (or near-complete) basis set this can be complicated by the basis-set superposition error (BSSE), necessitating a counterpoise correction using either the full dimer basis set (Boys & Bernardi, 1970 ▸) or approximations to this correction such as the geometric counterpoise correction (GCP) (Kruse & Grimme, 2012 ▸).

We have previously demonstrated the value of an alternative approach to computing dimer energies that involves fitting scale factors to components from a QM method based on the Hartree–Fock (HF) interaction energy (Su & Li, 2009 ▸) to accelerate the determination of these interaction energies (Turner *et al.*, 2014 ▸; Mackenzie *et al.*, 2017 ▸) and made this available through the *CrystalExplorer* software (Spackman *et al.*, 2021 ▸), where it has found widespread use, particularly in the field of crystal engineering. This model avoids the full dimer (*AB*) SCF through approximating the change in mol­ecular orbitals (MOs) from *A* interacting with *B* via superposition of the two wavefunctions, along with a single orthogonalization step for the orbitals to respond to one another. This model, when coupled with scaling coefficients and force field-like terms for polarization and dispersion, has proved to be an effective and useful model for intermolecular interactions to aid in rationalizing molecular packing (Tan *et al.*, 2019 ▸), mechanical slip planes (Wang & Sun, 2018 ▸), halogen bonding (Brammer, 2017 ▸) and even prediction of crystal growth (Spackman *et al.*, 2023 ▸).

Despite their overall success, the previous models (CE-HF and CE-B3LYP) suffered from several primary (technical) shortcomings:

(i) The choice of the 6-31G(d,p) basis for the more accurate CE-B3LYP model prevented its application to many heavier elements.

(ii) The global dispersion model D2 (Grimme, 2006 ▸) does not depend on the monomer wavefunctions, which limits its accuracy.

(iii) The use of free atomic polarizabilities was a more extreme approximation, as such quantities change significantly when the atom is part of a molecule.

It is not only timely to address some shortcomings of the previous method, but also to highlight the value that the *CrystalExplorer* model energies, and other models such as the density functional tight-binding (DFTB) method GFN2-xTB (Bannwarth *et al.*, 2019 ▸), can provide in our classification and understanding of intermolecular interactions. Characterization of the strengths, weaknesses and range of applicability of these models, and making available a cohesive implementation to use both in the context of *CrystalExplorer* and on high-performance computing systems, is our fundamental purpose here. Thus in this work we aim to address these issues, while producing a model that is more accurate and transferable wherever possible.

To the above end, the interaction model, along with its software implementation, has been updated to incorporate the use of effective core potentials (ECPs, see Section S2.2 in the supporting information) which model core electrons in heavier elements. This not only increases the speed of the calculations for heavier elements, but enables the use of basis sets parameterized for use with ECPs, for example the Ahlrichs def2 basis sets (Weigend & Ahlrichs, 2005 ▸), which we make use of in this work. Furthermore, the previously used D2 dispersion model (Grimme, 2006 ▸) has been replaced with the theoretically motivated exchange-hole dipole model (XDM) (Johnson & Becke, 2006 ▸; Becke & Johnson, 2007 ▸; Otero-de-la-Roza & Johnson, 2012*b* ▸). This yields molecule-dependent dispersion parameters while also facilitating an elegant solution for atom-in-molecule polarizabilities, and is itself a drastic improvement in the treatment of dispersion.

## 2. Methods

### 2.1. Reference values used in the fitting procedure

In order to fit and evaluate new models, a modified version of the training set of molecular crystals used in the previous CE model fitting (Mackenzie *et al.*, 2017 ▸) was chosen. The revised set consists of 1157 molecule/ion pairs extracted from 147 organic, inorganic and organometallic/metal–organic molecular crystal structures, incorporating atoms up to and including bromine, and also iodine and xenon. For each molecular crystal structure, molecule/ion pairs were obtained by generating a cluster of nearest-neighbour interactions around each symmetry-unique molecule/ion in the crystal. A complete list of the crystal structures, including Cambridge Structural Database (CSD) refcodes (Groom *et al.*, 2016 ▸) and Inorganic Crystal Structure Database (ICSD) identifiers (Levin, 2020 ▸), is available in the supporting information.

Reference ωB97M-V/def2-QZVP interaction energies were then calculated via evaluation of the full dimer SCF energy, subtracting the monomer energies (in the monomer basis). The ωB97M-V method (Mardirossian & Head-Gordon, 2016 ▸) was selected because of its general accuracy and transferability across many problems, including non-covalent interactions (Najibi & Goerigk, 2018 ▸). The large def2-QZVP (Weigend *et al.*, 2003 ▸; Weigend & Ahlrichs, 2005 ▸; Peterson *et al.*, 2003 ▸) basis set was chosen to minimize BSSE where possible, while still being computationally feasible for dimers comprising hundreds of atoms. All calculations were performed using the *ORCA* software suite (Version 5; Neese, 2022 ▸). After evaluating reference energies, pairs with a significant amount of charge transfer were removed from the training set. This was deemed necessary since charge transfer is not an aspect we are attempting to model in this work, and so inclusion of values where this is a significant contribution to the interaction energy would only serve to confound the results of the fitting procedure(s) and error estimates. Furthermore, particularly in the context of molecular crystals, the charge transfer occurring in a gas-phase dimer does not necessarily correspond well to that present when the dimer is embedded in a crystalline environment with competing charge-transfer possibilities. Significant charge transfer, where a large portion of an electron has moved from one monomer to another, was, for our purposes, defined as 

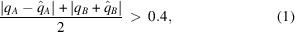

where *q*_*A*_ is the resulting Löwdin net charge on monomer *A* in the dimer calculation and 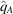 is the formal charge on monomer *A* in the monomer calculation (along with the corresponding definitions for monomer *B*). This resulted in the removal of 24 interaction pairs, all from the groups of organic salts and organic salt solvates.

The final set consists of 221 neutral organic pairs, 276 neutral closed-shell organometallic/metal–organic pairs, 341 pairs from organic salts and 319 organic salt solvates; a full listing of the crystal structures used is available in Table S1 in the supporting information. As in our previous work, it is our belief that the training set is well balanced, incorporating a wide range of typical atom environments and interaction types, and is robust enough that removal or addition of a few structures will have a minimal effect on the outcomes.

### 2.2. Fitting procedure

The energy model utilized throughout this work can be expressed as follows: 

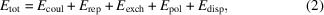

where the subscripts tot, coul, rep, exch, pol and disp indicate total, Coulomb, repulsion, exchange, polarization and dispersion components of the energy, respectively. Additional details of the evaluation of these terms are provided in the supporting information in Section S2. In previous work, the term *E*_exch-rep_ = *E*_rep_ + *E*_exch_ was used in place of expressing the two components separately. In that work, the *E*_exch-rep_ term was denoted simply as *E*_rep_, but the notation has been changed here to better express that it is a combination of the HF exchange term and repulsion due to MO changes, rather than a pure repulsive term. Likewise, the notation *E*_coul_ has been utilized here rather than *E*_ele_, although they are the same term.

The previous CE energy models utilized four empirical parameters, one scale factor for each term *E*_coul_, *E*_exch-rep_, *E*_pol_ and *E*_disp_. Although we can simply fit the same parameters for the model(s) pursued in this work, we have instead examined the possibility of separately scaling the exchange and repulsion terms *E*_exch_ and *E*_rep_.

Likewise, there are free parameters in the XDM model that may be fitted (see Section 2.4). As preliminary results and examination showed that fitting these XDM parameters, in addition to other scale factors, did not significantly improve the quality of the fit, we elected not to fit them, instead choosing simply to use values of *a*_1_ = 0.65 and *a*_2_ = 1.70 Å, which are relatively close to those utilized for the B86bPBE-25 hybrid by Price *et al.* (2023 ▸). Similarly, preliminary exploration of other terms outlined in Section 2.5, with (or without) their own scale factors, demonstrated little improvement to the fit.

Since the introduction of more parameters (and terms), as described above, was not found to give a systematic improvement to the quality of the fit to our training data – a strong indication that overfitting is occurring – three possible models were evaluated:

(i) CE-1p, a global single-parameter fit for the repulsion term and polarization term (*i.e.* *k*_rep_ = *k*_pol_, using the same parameter regardless of wavefunction source).

(ii) CE-2p, a two-parameter fit for the exchange-repulsion term and polarization term, fitting *k*_exch-rep_ and *k*_pol_ separately, and using the same parameters regardless of wavefunction source.

(iii) CE-5p, a five-parameter fit with separate scale factors *k*_coul_, *k*_rep_, *k*_exch_, *k*_pol_ and *k*_disp_.

The main motivation for examining these particular fits were the following theoretical arguments. The long-range behaviour for *E*_coul_ should be exact, along with it typically being the dominant term (especially for charged systems). Likewise, the XDM dispersion term is well defined and theoretically sound, while the weakest terms theoretically are the polarization treatment (which is only exact for spherical atoms, which is not the case here) and the *E*_rep_ term which *would* be exact if the full dimer SCF were performed. However, since we are only orthonormalizing the MOs for the monomers in the dimer systems, this ought to overestimate the repulsion as we are not allowing the MOs to relax after the initial change in the dimer environment.

### 2.3. Monomer wavefunctions

There are two primary considerations when selecting a source of monomer wavefunctions for the proposed model energies: choice of method, be it HF, density functional theory (DFT) or otherwise, and choice of basis set. All of the options considered in this work utilize the def2-SVP (Weigend & Ahlrichs, 2005 ▸) basis set. This was chosen because of its moderate cost (no diffuse functions, but retaining polarization functions) and wide support for elements across the periodic table (H to Rn), with corresponding ECPs (Peterson *et al.*, 2003 ▸) for heavier elements.

When considering candidate computational methods, we would intuitively expect to see systematic improvement from better molecular wavefunction sources, corresponding to the generally accepted levels of theory in the broader computational chemistry context. As such, we evaluated several different wavefunction sources: HF, LDA (SVWN5) (Dirac, 1930 ▸; Bloch, 1929 ▸; Vosko *et al.*, 1980 ▸), BLYP (Becke, 1988*b* ▸; Lee *et al.*, 1988 ▸; Miehlich *et al.*, 1989 ▸), B3LYP (Stephens *et al.*, 1994 ▸), ωB97x (Chai & Head-Gordon, 2008 ▸) and ωB97M-V (Mardirossian & Head-Gordon, 2016 ▸). HF and LDA were chosen because of their ubiquity and simplicity, and because they serve as good reference points for understanding the contributions of relative errors and are well characterized. The other choices represent different ‘rungs’ on the ladder of DFT accuracy: BLYP remains a popular GGA (generalized gradient approximation) density functional approximation, with generally good accuracy for non-covalent interactions when combined with appropriate dispersion corrections (Goerigk *et al.*, 2017 ▸). Likewise, dispersion-corrected B3LYP (hybrid-GGA) has demonstrated accuracy for non-covalent interactions and is the wavefunction source used in the CE-B3LYP model from our previous work (Mackenzie *et al.*, 2017 ▸). Finally, as previously mentioned, ωB97M-V has been widely demonstrated to be a reliable functional for a variety of chemical problems (most importantly non-covalent inter­actions) (Najibi & Goerigk, 2018 ▸), and as such it was chosen for reference calculations for the CE fitting data set. We also investigated ωB97x as a reference point, as it is more widely implemented (range-separated hybrid GGA), and given that the default in *e.g.* *ORCA* is to use the VV10 (Vydrov & Van Voorhis, 2010 ▸) non-local correlation functional as a post-SCF correction only, there should be only small differences in the MOs between the two methods.

### 2.4. XDM dispersion implementation

The XDM dispersion model was implemented and tested for correctness against the existing implementation in the *postg* program (Otero-de-la-Roza & Johnson, 2013 ▸; Kannemann & Becke, 2010 ▸). The present implementation makes use of the Becke–Roussel (Becke & Roussel, 1989 ▸; Proynov *et al.*, 2008 ▸) density functional approximation for the exchange-hole calculations, with the same sort of grids used in numerical integration for DFT approximations in the code, namely the Becke integration scheme (Becke, 1988*a* ▸) using Lebedev (1976 ▸) angular quadratures and the radial quadrature scheme from Lindh *et al.* (2001 ▸).

For the Hirshfeld (1977 ▸) partitioning part of the model, atomic wavefunctions from Koga *et al.* (1993 ▸, 2000 ▸) were used for the spherically symmetric gas-phase atomic electron densities. The grids used in the numerical integration were generated using the typical Becke partitioning scheme (Becke, 1988*a* ▸), as the implementation could use the existing framework already present in DFT calculations.

Atomic polarizabilities utilized throughout were the same as those used in the previous CE models (Turner *et al.*, 2014 ▸), and the resulting in-molecule polarizabilities are used internally in the XDM methodology as follows: 

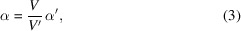

where α and *V* are the in-molecule polarizability and Hirshfeld volume, respectively, and α′ and *V*′ are the corresponding quantities for the free atom. This is a simple approximation that accounts for the (typically contracted) atomic volume in a molecule.

As formulated, the XDM dispersion model has two free parameters associated with the damping at close range, *a*_1_ and *a*_2_. The energy is formulated as follows:

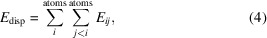




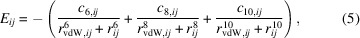




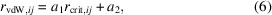




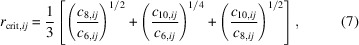

where *i* and *j* are atoms, *c* are the coefficients specific to the atom pair *ij* and *r* is the interatomic distance. Subscripts vdW and crit indicate van der Waals and critical radius, respectively. In essence, *a*_1_ is a scale factor for the critical radius where all three terms (1/*r* ^6^, 1/*r* ^8^ and 1/*r* ^10^) are equal, and *a*_2_ is a constant in distance units.

The above expression is, of course, the total dispersion energy for a system, not the interaction energy between two molecules. The interaction energy *E*_int_ can itself be calculated either by finding the corresponding energy terms for both monomers and the dimer, 

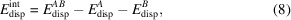

or, equivalently, by simply summing only over *i* ∈ *A* and *j* ∈ *B* in equation (4).

One consideration here, given that we are not performing an SCF for the dimer *AB*, is in the choice of XDM coefficients – we can either re-use the coefficients calculated for the monomers *A* and *B* (referred to as the monomer dispersion model), or calculate the coefficients for the concatenated dimer wavefunction and use equation (8) (referred to as the dimer dispersion model in this work).

### 2.5. Other considerations

As briefly mentioned when discussing the fitting procedure, we explored various possible low-cost enhancements and modifications for the accuracy of the energy models in this work. Specifically, we evaluated the possible improvement in performance when fitting the model variants against the S66x8 (Řezáč *et al.*, 2011*a* ▸,*b* ▸; Brauer *et al.*, 2016 ▸) benchmark set, separately incorporating the geometric counterpoise correction (GCP) term (Kruse & Grimme, 2012 ▸) including the accompanying small basis-set correction utilized in the HF-3c model (Sure & Grimme, 2013 ▸), fitting both the XDM *a*_1_ and *a*_2_ parameters, using the larger def2-TZVP basis set, using a DFT interaction model (see Section S2.1) and fitting a force field-like repulsion term similar to that found in the DREIDING model (Mayo *et al.*, 1990 ▸) to correct interaction energies at short distances.

Preliminary results demonstrated that none of the above modifications systematically improved the performance of the model energies against their training set when compared with simply re-fitting scale factors in either the CE-B3LYP or CE-5p models fitted to the same data. In other words, the only improvements were attributed to the fitting of the parameters to the set being evaluated, rather than the introduction of the different terms. As such, none of these modifications were incorporated into the models proposed in this work.

### 2.6. Rotation of molecular orbitals using pure spherical basis sets

Since the model shown in this work, and the previous CE models, start with isolated monomer wavefunctions, it is expedient to re-use the same wavefunction (after rotation and translation to the new position) rather than recalculate each monomer multiple times, particularly in the context of mol­ecular crystals where there is typically only one (or a handful of) symmetry-unique molecules.

The above rotation was previously implemented only for wavefunctions in a Cartesian spherical harmonic basis-set convention, but has now been extended to direct rotation of ‘pure’ spherical harmonic basis sets. This has been implemented using the already known recurrence relations (Ivanic & Ruedenberg, 1998 ▸). Facilitating the use of pure spherical basis sets is valuable as it allows for the use of other QM programs (such as *ORCA*) which may only support the use of pure spherical basis sets, and may help to reduce computation times where many higher angular momentum (d, f, g *etc.*) functions are included.

### 2.7. Reference lattice energies for molecular crystals

In previous work we highlighted the surprising success and accuracy of the CE-B3LYP model in predicting lattice energies of molecular crystals when using direct summation. To compare the models developed in this work against accurate reference data, along with the previous model, we now examine the performance on the X23 (Reilly & Tkatchenko, 2013 ▸) benchmark set, specifically using the revised reference energies in the X23b set (Dolgonos *et al.*, 2019 ▸).

We have examined the predicted lattice energies for both the experimental geometry (after normalizing *X*—H bond lengths to average crystallographic values) and the PBE0+MBD/light optimized geometries given by Dolgonos *et al.* (2019 ▸).

## 3. Results and discussion

### 3.1. Fitting results

The previous model fitted four parameters, the scale factors *k*_ele_, *k*_exch-rep_, *k*_pol_ and *k*_disp_. In this work we examine the CE-5p model which separately fits five parameters (splitting the exch-rep term, resulting in separate *k*_exch_ and *k*_rep_ parameters), the CE-2p model with two parameters (the scale factors *k*_exch-rep_ and *k*_pol_) and the single-parameter model CE-1p with only *k* = *k*_rep_ = *k*_pol_. In part, the last two fits are motivated by the use of a new (and more accurate) dispersion term, and it was thought that fixing the corresponding scale factors for this and the Coulomb term to unity (*i.e.* unscaled) would be a reasonable starting point and might result in a more transferable fit to other methods and/or basis sets.

**Figure 1:**
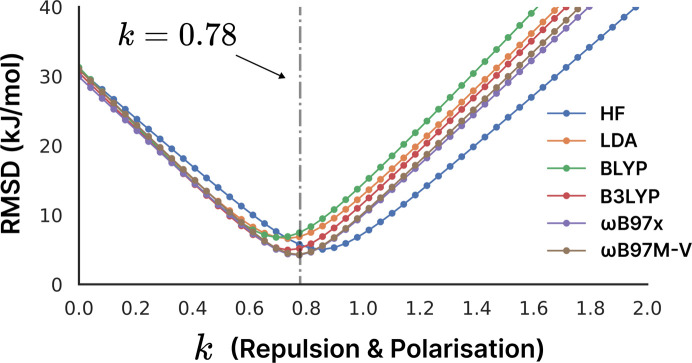
Error distributions for HF, LDA, BLYP, B3LYP, ωB97x and ωB97M-V methods over a range of *k* = *k*_rep_ = *k*_pol_ values. While there are clearly some differences between the models, it should be seen that the minimum for ωB97M-V at *k* = 0.78 (highlighted by the grey dot-dashed line) still produces good results for the other models.

To examine transferability, once all energy terms were evaluated for the training set (see Section S2 for full details), rather than simply performing a least-squares fit for the CE-1p and CE-2p models to minimize the errors, we examined the error over a range of *k* in the case of CE-1p, and a distribution of *k*_exch-rep_ and *k*_pol_ in the case of CE-2p. The results for this process are shown in Fig. 1 ▸ for the case of CE-1p and in Fig. S2 for the case of the CE-2p model.

In the case of CE-1p, there is substantial agreement between different wavefunction sources for the optimal value of the fitted *k* parameter, with a minimum in the case of ωB97M-V at 0.77850434…, *i.e.* 0.78 (to 2 d.p.). The only method with a significantly shifted minimum value is HF, but even there the difference between the RMSD of 5.0 kJ mol^−1^ for the optimal value (*k* = 0.86) versus 5.7 kJ mol^−1^ for the HF model is less than 1 kJ mol^−1^.

For the case of CE-2p, all methods yield minima in a similar area and there is a region of the two-parameter space where the error is relatively flat (plots are provided in Fig. S2). Given that, intuitively, ωB97M-V should be the most accurate method against the reference (being the same method as used in the reference), we elected to use two parameters from the minima of this approach, located at *k*_rep_ = 0.485 and *k*_pol_ = 0.803. However, it must be pointed out that the agreement between the two-dimensional minima in CE-2p is much worse than in the one-dimensional case of CE-1p, which indicates that its transferability to different molecular wavefunction sources is likely to be inferior.

The five-parameter model (CE-5p) was also examined, with scale factors fitted using least squares, and the resulting coefficients are given in Table 1 ▸. For the five-parameter fit, values for all scale coefficients were fixed in the range [0, 3], with the exception of *k*_rep_, which was bounded in the range [0.6, 3]. These boundaries were introduced because of the potential for over-fitting – it was found that freely optimizing the parameters introduced negative scale factors or scale factors close to zero, with very small improvements to the errors for the model. Based on the exploration of the parameter space for the CE-1p model, *k*_rep_ ≥ 0.6 was chosen to allow enough flexibility to alter the *k*_rep_ parameter while constraining the fit to more (theoretically) reasonable parameters. It can be seen here that the Coulomb scaling coefficient is very close to unity for all wavefunction sources, which is rationalized by the number of charged systems in the training set, where this will be the overwhelmingly dominant term for most of them.

**Figure 2:**
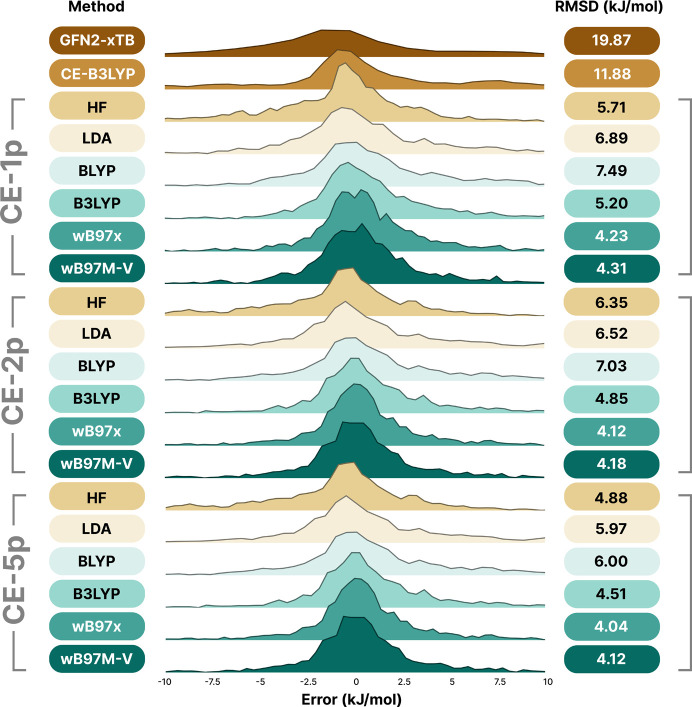
Kernel density estimate plots, showing the errors for the predicted energies versus the reference (ωB97M-V/def2-QZVP) dimer interaction energies in the training set when using the previous CE-B3LYP model, and GFN2-xTB, along with the fitted CE-1p, CE-2p and CE-5p using the six wavefunction sources explored in this work (top to bottom): HF, LDA (SVWN5), BLYP, B3LYP, ωB97X, ωB97M, with RMSD values provided to the right-hand side of each plot. Each wavefunction source has been shifted on the *y* axis to facilitate visual inspection of the distributions.

Error distributions for the training set are shown in Fig. 2 ▸, where it is notable that, for all sources other than HF, the five-parameter fit produces only negligible improvements in overall energy errors relative to the transferable one and two-parameter fits. This, coupled with the need to introduce constraints, is indicative that it is likely we are in the realm of overfitting. To examine this further, we must look outside the training set to evaluate the behaviour against external reference data.

### 3.2. S66x8 set

The primary use case for the proposed energy models, and indeed the major use case for the previous models, is intermolecular interactions in organic molecular crystals, *i.e.* intermolecular interactions between neutral organic species. To this end, the S66x8 benchmark set (Řezáč *et al.*, 2011*a* ▸,*b* ▸; Brauer *et al.*, 2016 ▸) constitutes an excellent test set to evaluate the performance of the fitted models over a range of intermolecular interaction distances and understand its behaviour for different interaction types. The overall results are shown in Table 2 ▸. Unsurprisingly, the previously fitted model (CE-B3LYP) performs very well for neutral systems, just as it did on the training set and in previous work. Likewise, across all fits, the B3LYP, ωB97X and ωB97M-V wavefunction sources are significantly better choices than HF, LDA and BLYP. This is not a surprising result, but it is consistent with intuition when it comes to the superior accuracy of (hybrid) DFT functionals.

Perhaps the most important conclusion from the results for this test set is that the five-parameter fits are only marginally better in performance (or sometimes worse) than the two-parameter fits. The performances for ωB97x and ωB97M-V are almost identical, but this is unsurprising when we consider how similar the models are in terms of their wavefunctions (the VV10 non-local correlation functional has only been applied as a post-SCF correction).

**Figure 3:**
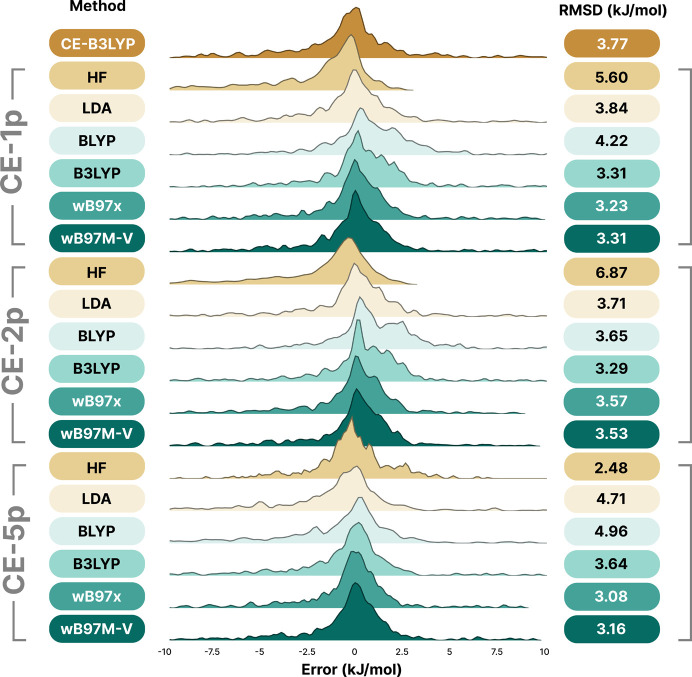
Kernel density estimate plots for the S66x8 set using the CE-1p, CE-2p and CE-5p fits, the previous CE-B3LYP model and the six wavefunction sources used in this work (top to bottom): HF, LDA (SVWN5), BLYP, B3LYP, ωB97X, ωB97M-V, with RMSD values provided on the right-hand side of each plot. Each wavefunction source has been shifted on the *y* axis to facilitate visual inspection of the distributions.

Similar to the results for the training set in the previous section, in Fig. 3 ▸ it is apparent that using more parameters does not necessarily lead to wholesale improvement of the reliability of the model, and where it does the improvement is relatively minor when considering potential applications to non-covalent interactions. The best result for CE-5p, for example, was actually HF with an RMSD of 2.5 kJ mol^−1^, versus the best result for CE-1p being ωB97X with an RMSD of 3.2 kJ mol^−1^. This is not totally insignificant, but when put in the context of the training data and the kinds of interactions that are present in the S66x8 data set, and the difference between five parameters (that may be less transferable) and only a single parameter, we believe the trade off of around 0.5 kJ mol^−1^ (which largely corresponds to a shift in mean error) is worthwhile.

With few exceptions (BLYP and HF in some cases), the error distributions are largely symmetric about zero, though there are longer and denser tails in the over-binding direction, which may be explained through the trends at different intermolecular separations in the S66x8 set. Once again, in this context the B3LYP, ωB97M-V and ωB97x methods are clearly superior in their reliability, particularly for the more transferable CE-1p model.

It is worth comparing our results with the recently published ωB97X-3c composite method (Müller *et al.*, 2023 ▸), which has also been evaluated against the S66 set (*i.e.* the subset of the S66x8 set with separation scale factor *r* = 1.0). Müller *et al.* (2023 ▸) gave ωB97X-3c an MAD of roughly 1.2 kJ mol^−1^ over these interactions, compared to, say, the corresponding ωB97X variant of the CE-1p model at roughly 1.8 kJ mol^−1^ (the corresponding value for CE-B3LYP is 2.5 kJ mol^−1^). Likewise, natural comparison methods for the approach are the SAPT(DFT) and SAPT0 methods, for which the corresponding MAD values, taken from Xie *et al.* (2022 ▸), are 1.4 kJ mol^−1^ for SAPT(DFT) and 4.1 kJ mol^−1^ for SAPT0. A full list of the MAD values for the S66 set is provided in Table S4. We believe this represents excellent agreement considering the relative cost of the methods, with our model not requiring an SCF for the combined dimer wavefunction.

### 3.3. Trends in error versus separation distance

In the context of molecular crystals, it is of course important for dimer interaction energies to be accurate over a range of intermolecular separations. Furthermore, characterization of the kinds of errors and their trends with distance and interaction type gives insight into where models are accurate and where their accuracy may be limited, be it systematic or otherwise.

**Figure 4:**
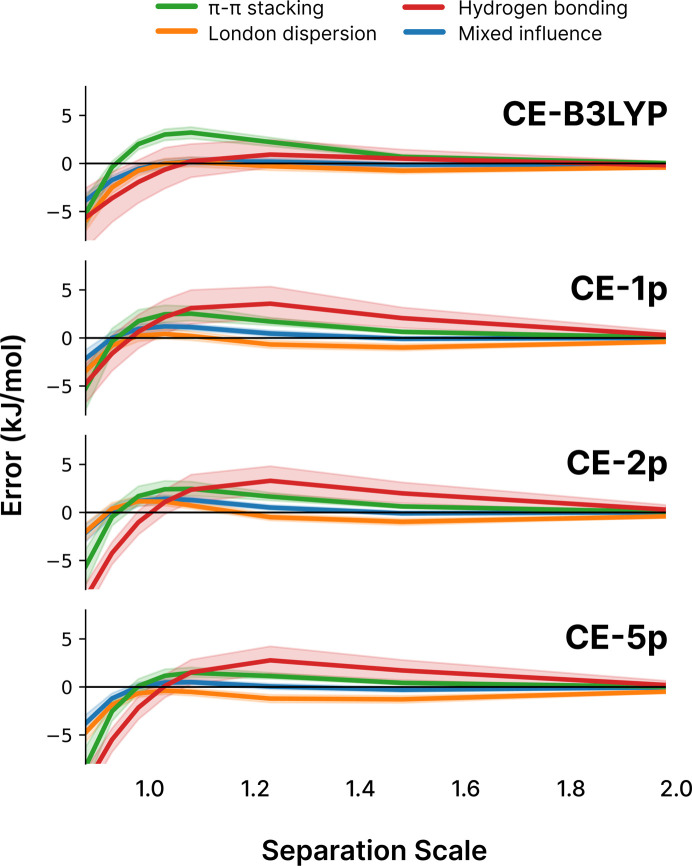
Trends in errors across different scaled separations for the S66x8 benchmark set when using different models in this work: CE-B3LYP (top), and CE-1p (upper middle), CE-2p (lower middle) and CE-5p (bottom) for the B3LYP wavefunction source. The solid lines indicate the mean error value, while the shaded regions represent the 95% confidence interval. The separation scale is relative to the equilibrium separation, *i.e.* a value of 1.0 is at equilibrium, while a value of 0.9 is 90% of the equlibrium separation. Values between explicitly calculated separations (0.9, 0.95, 1.0, 1.05, 1.1, 1.25, 1.5, 2.0) have been linearly interpolated in order to show the behavioural trends.

Careful examination of Fig. 4 ▸ shows that the distribution of errors surrounding the mean error when using the CE-5p fit is marginally narrower, but it appears that the CE-5p fit is indeed over-fitted, as its systematic over-binding at closer-than-equilibrium intermolecular separations (0.9, 0.95 in the separation scale) is more extreme than for both the previous CE-B3LYP model and the CE-1p model. This trend, coupled with the marginal difference in overall performance for the CE-1p fit versus the CE-5p fit (3.3 kJ mol^−1^ for CE-1p using B3LYP, versus 2.5 kJ mol^−1^ RMSD for the best CE-5p model which used HF) is, in our judgement, enough to recommend usage of the CE-1p model, with only a single free parameter that is clearly transferable across wavefunction sources.

Errors at closer-than-equilibrium separations are of particular relevance to high-pressure studies of molecular crystals, and previous work (Eikeland *et al.*, 2017 ▸) has shown that the CE-B3LYP model demonstrated significant errors compared to counterpoise-corrected B3LYP calculations on dimers in hydroquinone clathrates of methanol and acetonitrile. This was at least partly attributed to the *k*_exch-rep_ scaling coefficient affecting repulsion at close separations. To examine this behaviour with the now larger repulsion scaling parameter for the CE-1p model relative to the previous CE-B3LYP model (*k* = 0.78 versus *k*_exch-rep_ = 0.6177), we evaluated dimer inter­action energies for the same systems as Eikeland *et al.* (2017 ▸) when using ωB97M-V/def2-svp as the wavefunction source. The results of these calculations, comparing CE-B3LYP, CE-1p and GFN2-xTB, are summarized in Figs. S3 and S4. The results are mixed: some systems show improvements over the previous CE-B3LYP model, others do not. The CE-1p model cannot be said to be an improvement in this regard over the old model, but neither can it be said to be worse. This is indicative that, despite the previous discussion by Eikeland *et al.* (2017 ▸), the errors are not solely associated with the repulsion scale factor, as there is a significant interplay between the dispersion damping coefficients as well. Ultimately, if we wish to have an improved model at closer intermolecular separations then, at a minimum, the training data set would need to be re-examined, along with changes probably being required in the assumptions of the model (*e.g.* a departure from global scaling parameters), which is outside the scope of this work. However, it is worth emphasizing that, as interactions get stronger at closer separations, we should expect that the errors get larger for almost any method, and that the present model, where we are assuming relatively little deformation in the molecular electron density, will eventually break down at close enough separations where significant overlap between monomer wavefunctions becomes a reality.

## 4. Lattice energies for molecular crystals

The previous CE models, particularly CE-B3LYP, have already been successfully applied to the prediction of lattice energies for neutral molecular crystals via the direct summation technique. Prediction of such lattice energies is particularly challenging due to the interplay of intermolecular interactions over a variety of distances where systematic errors will tend to manifest themselves largely as wrong absolute values for the lattice energies. At present, the X23 benchmark set (Reilly & Tkatchenko, 2013 ▸; Dolgonos *et al.*, 2019 ▸), itself an extension of the C21 set (Otero-de-la-Roza & Johnson, 2012*a* ▸), constitutes the most reliable and robust set of reference values for molecular crystal lattice energies.

Table 3 ▸ shows the resulting error statistics in lattice energies for the models explored in this work compared with those for the CE-B3LYP model. Optimized geometries that were previously used in the X23 revised benchmark (Dolgonos *et al.*, 2019 ▸) were used, as well as experimental geometries with normalized hydrogen-bond lengths; the results for the experimental geometries are available in Table S7. When interpreting these data, it is worth noting that the standard error (σ) for sublimation enthalpies is typically in the region of 4 kJ mol^−1^ (Chickos, 2003 ▸) and the correction procedure involving vibrational contributations to the sublimation enthalpy will further introduce its own errors. It is also worth highlighting that six of the molecules in the X23 set are present in the training set, namely urea (UREAXX), benzene (BENZEN01), imidazole (IMAZOL13), formamide (FORMAM02), succinic acid (SUCACB09) and uracil (URACIL). However, the training set is based only on the nearest dimers (rather than the dozens or hundreds of energies contributing to the lattice energy) and uses a different reference method than in the reference X23 data.

It can be seen in Table 3 ▸ that the errors for different models are nowhere near as uniform as they were for the S66x8 benchmark set. This should not be surprising – any systematic over- or under-estimation for interactions will be amplified when summing across many interactions/dimer pairs. The transferable single-parameter model demonstrates excellent performance and transferability across different wavefunction methods, though the success is significantly diminished when examining the HF results.

There is little doubt that the accuracy of the models presented here, when using direct summation for the lattice energies, is reliant on cancellation of errors (though the same may be said for all other methods). Nevertheless, the results for the CE-1p model using B3LYP, with an MAD of only 3.6 kJ mol^−1^, compares very favourably with the current state of the art (Price *et al.*, 2023 ▸), where the *best* method (a composite of B86bPBE-25-XDM//B86bPBE-XDM approaches including a basis-set correction) reported had an MAD of 2 kJ mol^−1^. For comparison, the same work reported PBE0-MBD giving an MAD of 3.5 kJ mol^−1^ when using the ‘Tight’ basis set.

**Figure 5:**
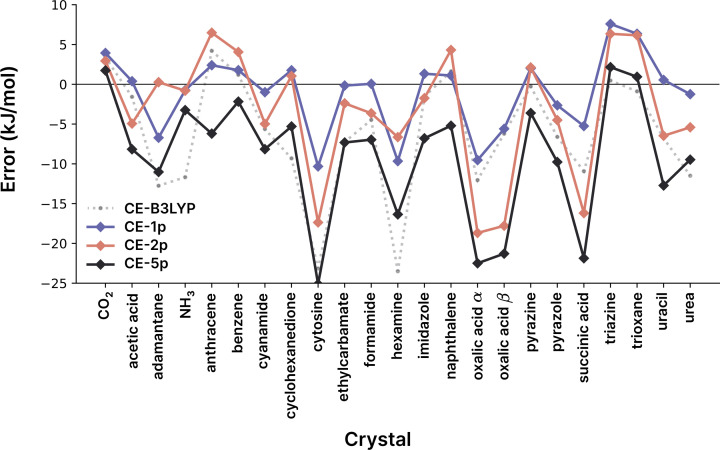
Errors in predicted X23 energies (difference from reference values) for the CE-B3LYP (grey dotted line) and the CE-1p (blue), CE-2p (orange) and CE-5p (black) models using B3LYP/def2-SVP as the wavefunction source. The overall error statistics for all models are given in Table 3 ▸. All values here are calculated using the PBE0+MBD/light optimized geometries used by Dolgonos *et al.* (2019 ▸). For the corresponding values in the experimental geometries see Table S7.

The relative behaviour of the CE-1p, CE-2p and CE-5p models when using the B3LYP functional against CE-B3LYP may be seen in Fig. 5 ▸, which indicates that the single-parameter model is an improvement over the alternatives across almost every system. The results for cytosine indicate that there may be some systematic error in the models, which merits further investigation into polarization as discussed in the next sub-section.

### 4.1. Crystal polarization effects

There are several sources of possible error in the direct summation method used here for lattice energies, with one of the more significant sources of error being the treatment of polarization. In a crystalline environment, the framework utilized in the CE energy models assumes that the polarization contributions are pairwise additive. However, the true polarization energy is fundamentally many-body in nature because of the quadratic dependence on magnitude of the electric field:

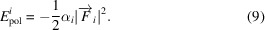

Here *i* is the *i*th atom, α_*i*_ is its polarizability, 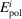 is the polarization energy for this atom and 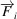 is the electric field at atom *i* from its environment, either from the neighbouring monomer (in the case of a pair) or from the crystal environment. While the field is just the pairwise sum over neighbours, the square of this quantity is no longer separable.

**Figure 6:**
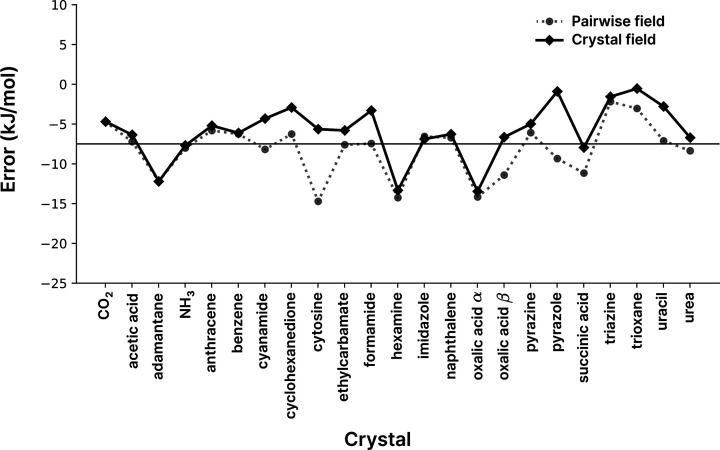
Errors in predicted X23 energies (difference from reference values) for the CE-1p method using B3LYP/def2-SVP as the wavefunction source when using two different polarization models. Here ‘Pairwise field’ corresponds to the usual method of calculating the polarization term for the lattice energy, *i.e.* each pair is calculated individually, whereas ‘Crystal field’ evaluates the electric field from all neighbours at the atomic positions of the symmetry-unique molecule(s) in the crystal. See equation (9).

The practical difference in the case of crystals may be seen in Fig. 6 ▸, where the sum of pairwise polarization terms overestimates the polarization energy of the crystal, especially for cytosine. In contrast, summing the field experienced by a monomer over all interactions in the crystal, before computing the polarization energy, significantly improves most energies for the X23 data set. It should be noted that there is a further effect of using gas-phase wavefunctions that may lead to underestimation of the polarization in comparison to the corresponding wavefunction in the solid state. Since gas-phase wavefunctions are used throughout the current procedure, we are likewise underestimating the polarization of the initial molecular wavefunction. These two factors will compete and we may (or may not) experience cancellation of errors, but this is an aspect that must be understood if, in future, molecule-in-crystal wavefunctions, for example, were to be used with this energy model to estimate lattice energies.

For now, we can examine the influence of evaluating the polarization energy using the theoretically more correct approach of summing over contributions to the field before computing the energy. This manifests in changes in the error statistics: the MAD value shifts to 4.4 kJ mol^−1^ (versus 3.6 kJ mol^−1^), largely due to the systematic shift in MSD to 2.2 kJ mol^−1^ (versus −1.0 kJ mol^−1^), while the RMSD only shifts to 5.3 kJ mol^−1^ (versus 4.8 kJ mol^−1^).

This suggests that if the goal is to predict relative lattice energies, as it so often is in the context of molecular crystals, then using a polarization term based on the electric field of the crystal should be considered. Indeed, there is a minor increase in error (less than 1 kJ mol^−1^) for the absolute values of the lattice energies compared with the reference values, but the errors are likewise more systematic which tends to improve the relative energies. Furthermore, in ionic systems the effects of the pairwise approximation for polarization are even greater. An illustrative example is cubic NaCl, which has zero net electric field at the ion positions, but when calculated pairwise there is an erroneous net polarization energy of around 400 kJ mol^−1^.

## 5. Conclusions

We have implemented, fitted and demonstrated the accuracy of a new single-parameter intermolecular interaction energy model, CE-1p, which is transferable across different wavefunction sources. The model constitutes an excellent accuracy/computational cost trade off, with an MAD of 2.0 kJ mol^−1^ across the S66x8 benchmark set, and near state-of-the-art performance when predicting lattice energies for the X23 benchmark set, with an MAD of 3.6 kJ mol^−1^, while using the simplest pairwise sum in real space. While there is little doubt that this model (and others) exhibits error cancellation, we believe it constitutes an efficient and accurate method for quantitative predictions of intermolecular interactions which may also be rationalized and understood through sensible separation of the energy into distinct contributions.

We have also examined the performance of the GFN2-xTB tight-binding model as a rapid method for similar purposes, with only relatively minor loss in accuracy for dimer energies in organic neutral systems. In particular, for neutral organic molecules (which constitute a large part of the chemical systems of interest in molecular crystals) using GFN2-xTB through visualization software such as *CrystalExplorer* provides near-instant feedback and allows for real-time exploratory evaluations, as in most cases it is one to two orders of magnitude faster than our energy model.

Our recommendations for the end user of these methods, who wishes to rationalize the intermolecular interactions in a molecular crystal, are as follows:

(i) First, calculate the interaction energies using GFN2-xTB, which is extremely low cost for pairwise interactions. It is particularly worth examining interactions beyond nearest-neighbours using such methods, as their long-range behaviour should be reliable. These can be visualized using energy frameworks (Turner *et al.*, 2014 ▸).

(ii) Calculate the same energies using the CE-1p model, using B3LYP, ωB97x or ωB97M-V functionals, depending on which is available in your QM software of choice.

(iii) If these models are not in agreement with respect to the relative strength of interactions, further detailed investigation with higher-level methods is warranted.

(iv) If lattice energies are of interest, strongly consider the polarity of the molecule(s) in your system, and whether or not the pairwise approximation for polarization is appropriate.

There are several avenues for further improvements and developments, including:

(i) The possibility of fitting a model using DFT exchange correlation rather than HF exchange, given the reduction in computational cost over evaluating the exact exchange matrices for the two monomers (*A* and *B*) and the better scaling speed-ups available for the Coulomb term, particularly if the overall accuracy of the model is not sacrificed.

(ii) Departing from global scaling parameters, particularly at close intermolecular separations, though this would be a significant change to the model.

(iii) Examination of the connection between the orthogonalized electron population (see Section S9) and the repulsion and exchange terms for development of low-cost approximations.

In summary, the new single-parameter CE-1p model is generally more accurate, is significantly more transferable (not only to other wavefunction methods but in its coverage of more of the periodic table, which enables application to more chemical problems) and has (qualitatively) a more sound theoretical basis than the existing CE-B3LYP model. Hence, we view it as an excellent replacement for the previous method which will no doubt find broad application across a variety of chemical problems, particularly in the domain of molecular crystals.

This work has been implemented and is already available in the open source software *occ* made available on GitHub (https://github.com/peterspackman/occ) The incorporation of the newly proposed CE-1p model, along with access to GFN2-xTB, will be available in a forthcoming release of *CrystalExplorer* and we are optimistic that both will be valuable additions for the community.

## 6. Related literature

For further literature related to the supporting information, see van Eijck & Kroon (1997 ▸) and Thomas *et al.* (2018 ▸).

## Supplementary Material

Additional details, figures and tables. DOI: 10.1107/S2052252523008941/fc5074sup1.pdf

Compressed archive of supporting data files. DOI: 10.1107/S2052252523008941/fc5074sup2.zip

## Figures and Tables

**Table 1:** Fitted scaling coefficients for the different components in the CE-5p model when using the six different wavefunction sources explored in this work, HF, LDA, BLYP, B3LYP ωB97X and ωB97M-V See equation (2) for the relevant energy expression. Note that *k*_rep_ values of 0.6 correspond to the fixed lower bound in the least-squares fitting procedure.

Coefficient	HF	LDA	BLYP	B3LYP	ωB97X	ωB97M-V
*k*_coul_	0.999	1.011	1.011	1.007	1.005	1.005
*k*_exch_	1.487	0.717	0.761	0.702	0.666	0.670
*k*_rep_	1.109	0.600	0.600	0.600	0.600	0.600
*k*_pol_	0.780	0.824	0.826	0.808	0.794	0.793
*k*_disp_	0.990	1.036	1.039	1.068	1.065	1.051

**Table:** 

		CE-1p					
Statistic	CE-B3LYP	HF	LDA	BLYP	B3LYP	ωB97X	ωB97M-V
MAD	2.4	3.3	2.6	2.6	2.1	2.0	2.0
MSD	−0.8	−3.1	−0.4	1.2	0.2	−0.5	−0.5
RMSD	3.8	5.6	3.8	4.2	3.3	3.2	3.3

**Table:** 

	CE-2p					
Statistic	HF	LDA	BLYP	B3LYP	ωB97X	ωB97M-V
MAD	3.7	2.2	2.3	2.1	2.1	2.1
MSD	−3.4	−0.5	0.9	−0.1	−0.8	−0.7
RMSD	6.9	3.7	3.7	3.3	3.6	3.5

**Table:** 

	CE-5p					
Statistic	HF	LDA	BLYP	B3LYP	ωB97X	ωB97M-V
MAD	1.7	2.6	2.8	2.0	1.7	1.8
MSD	−0.1	−1.7	−1.5	−0.8	−0.5	−0.4
RMSD	2.7	4.6	5.0	3.5	2.9	2.9

**Table:** All values here are calculated using the PBE0+MBD/light optimized geometries used by Dolgonos *et al.* (2019 ▸). For the corresponding values in the experimental geometries see Table S7.

		CE-1p					
Statistic	CE-B3LYP	HF	LDA	BLYP	B3LYP	ωB97X	ωB97M-V
MAD	7.3	18.4	4.3	4.7	3.6	5.2	5.9
MSD	−6.3	−18.4	−2.8	3.0	−1.0	−4.1	−4.9
RMSD	9.7	23.2	5.8	5.6	4.8	7.1	7.6

**Table:** 

		CE-2p					
Statistic	GFN-xTB	HF	LDA	BLYP	B3LYP	ωB97X	ωB97M-V
MAD	11.2	21.0	6.0	4.7	6.3	8.2	7.9
MSD	−7.6	−20.9	−4.2	1.3	−3.4	−6.7	−6.7
RMSD	15.2	27.6	7.7	5.6	8.4	11.2	10.9

**Table:** 

	CE-5p					
Statistic	HF	LDA	BLYP	B3LYP	ωB97X	ωB97M-V
MAD	5.4	12.0	13.2	9.5	7.3	7.4
MSD	−3.6	−11.5	−12.7	−9.1	−6.7	−6.8
RMSD	8.1	14.0	15.6	11.8	9.6	9.7
