# Supplementary material for: A transferable quantum mechanical energy model for intermolecular interactions using a single empirical parameter
Source: IUCrJ. 2023 Oct 31;10(Pt 6):754–65. doi: 10.1107/S2052252523008941 (PMC10619445; doi:10.1107/S2052252523008941)
Supplement: Supplementary file 1 [file m-10-00754-sup1.pdf]

IUCrJ

Volume 11 (2024)

Supporting information for article:

A transferable quantum mechanical energy model for intermolecular interactions using a single empirical parameter

Peter R. Spackman, Mark A. Spackman and Julian D. Gale

A transferable quantum mechanical energy model for intermolecular interactions using a single empirical parameter - Supporting Information

Peter R. Spackman,[a]* Mark A. Spackman[b] and Julian D. Gale[a]

[a]School of Molecular and Life Sciences, Curtin University, PO Box U1987, Perth, Western Australia 6845, Australia, and [b]School of Molecular Sciences, University of Western Australia, Perth 6009, Australia. E-mail: peter.spackman@curtin.edu.au

S1. Listing of additional files for Supporting Information

- `ce1p.csv`, `ce2p.csv`, `ce5p.csv`: interaction energies (including components, scaled total energies and reference values) for the training set in this work, separated by model according to filename.
- `dispersion_difference_wb97m.csv`: interaction energy (dispersion) data showcasing the difference between using the derived dimer parameters for XDM and the monomer parameters.
- `s66x8.csv`: interaction energies (including components, scaled total energies and reference values) for the S66x8 test set for all models in this work.
- `x23.csv`: total lattice energies (including components, scaled total energies and reference values) for the X23 test set for all models in this work, including crystal polarisation values (`cpol`) and the relevant monomer corrections to be applied.

S2. Description of the interaction energy model

The model utilises the two monomer wavefunctions Ψ_A, Ψ_B and the merged dimer wavefunctions Ψ_{AB} (the concatenation of the two monomer wavefunctions) and $\Psi^{\perp}_{AB}$

(the merged wavefunction after orthogonalisation of the molecular orbitals.

Typically the energetic terms in the CE model involve the expectation values of operators, i.e.

$$E_{\hat{V}} = \left\langle \Psi \middle| \hat{V} \middle| \Psi \right\rangle \tag{S1}$$

where, for example the exchange operator for monomer A, $\hat{K}$, would yield the exchange energy for Ψ_A:

$$E^A_{\hat{K}} = \left\langle \Psi_A \middle| \hat{K} \middle| \Psi_A \right\rangle \tag{S2}$$

The full exchange-repulsion term E_{rep} in our model is given by:

$$E_{\text{rep}} = (E_{\hat{K}} - E^A_{\hat{K}} - E^B_{\hat{K}}) + (E^{\perp}_{\text{core}} - E_{\text{core}} + E^{\perp}_{\hat{J}} - E_{\hat{J}} + E^{\perp}_{\hat{K}} - E_{\hat{K}}) \tag{S3}$$

where E_{core} denotes the energy of the core Hamiltonian, incorporating the kinetic energy $\hat{T}$, electron-nuclear attraction $\hat{V}_{\text{en}}$ potential operators.

S2.1. Use of DFT exchange-correlation rather than HF exchange

When we are utilising density fitting procedures, computation of the Coulomb matrix $\hat{J}$ (and thus the corresponding energy term, E_{coul}) is much more efficient than the exchange matrix $\hat{K}$, due to the separation of integrals. The energetic term for the exchange energy, $E_{\hat{K}}$, can be calculated using density functional theory (i.e. replaced with E_{xc}), where for pure (i.e. non-hybrid) DFs the equivalent energy term will be:

$$E_{\text{rep}} = (E_{\text{xc}} - E^A_{\text{xc}} - E^B_{\text{xc}}) + (E^{\perp}_{\text{core}} - E_{\text{core}} + E^{\perp}_{\text{J}} - E_{\text{J}} + E^{\perp}_{\text{xc}} - E_{\text{xc}}) \tag{S4}$$

It should be obvious that $E_{\text{xc}} \neq E_{\text{x}}$, but they are equivalent in their purpose for modelling, and result in a lower-cost interaction energy model where a larger basis

set (e.g. def2-TZVP rather than def2-SVP) may be used for relatively little additional computation time and, depending on the density functional approximation further incorporate some correlation effects.

S2.2. Interaction energy model incorporating ECPs

The Coulomb energy term in the pair interaction is given by:

$$E_{\mathrm{coul}} = E_{\hat{J}} + E_{\mathrm{nn}} + E_{\hat{V}_{\mathrm{en}}} \tag{S5}$$

where $\hat{J}$ is the Coulomb operator, the subscript nn represents nuclear-nuclear repulsion and $\hat{V}_{\mathrm{en}}$ is the nuclear attraction operator.

Since the effective core potentials are essentially modelling both core electrons and nuclear charge, their potential $\hat{V}_{\mathrm{ECP}}$ is included in the Coulomb term as follows

$$E_{\mathrm{coul}} = E_{\hat{J}} + E_{\mathrm{nn}} + E_{\hat{V}_{\mathrm{en}}} + E_{\hat{V}_{\mathrm{ECP}}} \tag{S6}$$

S3. Structures used in the Fitting Procedure

Table S1 contains a complete listing of the structures used to generate molecular dimers in this work.

Table S1. *CSD and ICSD refcodes of crystal structures used in the fitting procedure*

Group			
Neutral organic	Organic salt solvates	Organic salts	Organometallic/Metal-organic
MTHMAD13	ISIHIB	PAXNIN	NIDCAR06
AEPHOS02	HODPUM	PAMBZA03	JARNAS
JUPPAL	IDOPAT	DEZYEO	PMCCOC
NAPHQU	HORVAL	BEPTUN	VANPAB
IMAZOL13	JAMMIT	TUYKAB	CCRTOL
JAGREP	MUTWUT	LARASC20	CEFFOI
VITRUL	FOVCAV	HEZXAM	FURRAL
30501-ICSD	YEFVAH	YABFOY	SAYVOE
ARSACP02	ROJJEG	MATPHB	CPTIBP01
URACIL	GELSUM	RIRMUA	VAACAC02
FORMAM02	SIMRAI	LIHMAM	PESKEE01
FORMAC01	KOLDUL	BOGCAC	CPCHTI
SUCACB09	HURMOX	HISREG	ZOSWEJ
MEADEN05	UGULUF	MIBTOH	JAFNAG
PVVAWA01	VEMJII	ZOWWEP	CPMNCO01
BENZEN01	IRURAO	ANLINC02	DIGWUL
ACETYL02		FENYEC	ACACCS
UREAXX12		DUVCAZ	BENDAB
ACOKEI		CUZHEK	FOHCOU
LALNIN03		KUDFIA	BZCRCO
DCLBEN06		COXYIY	RUJBUT
JOYHUA		TRPPAC	TITODC10
201142-ICSD		NAASCB	QENJAV
PYRIDO04		MEDHOU01	TESBUO
201698-ICSD		DUBWAZ	TCBMNI
QEWXIA01		DUFBIP	GIHWIE01
VAMZIT01			PVVBAY01
NTRGUA03			COACAC03
200455-ICSD			COSRUX
DCLBEN07			NEZWEU
260950-ICSD			DADHIZ
EBIHIH			NCKLCN
ETHLEN10			CEHPIO01
ETHANE01			VIPGEG
DCLBEN03			HEPSOL
15318-ICSD			TROPSC
201693-ICSD			YABGAK
			DCPVCL
			ACACSC
			FOMKIB01
			NISALO01
			FEROCE27
			DBENCR10

Where hydrogen-bond lengths were normalised, the values used for each bond type are given in Table S2.

Table S2. *Bond lengths used for normalising terminal X−H bonds (e.g. in the X23 set for experimental crystal structures)*

Bond	Length (Å)
B−H	1.180
C−H	1.083
N−H	1.009
O−H	0.983

S4. XDM dispersion: pair parameters vs. monomer parameters

While the XDM method is a density-dependent dispersion correction, it is worth noting that for non-covalent interactions, where there is relatively small overlap between molecular orbitals of the two dimers, the derived parameters of the XDM method i.e., polarisabilities, atomic moments, atomic volumes and atomic free volumes will typically be relatively unchanged. This can be seen when comparing the dispersion interaction energy of dimers AB when calculating using only parameters from the monomer wavefunctions vs. those derived from the combined wavefunction. In the evaluated molecular pairs, the mean absolute percentage error is 3.72%, and as seen in Figure S1 90 % of values fall within a range of $(-0.70, 0.15)$ kJ/mol. Perhaps more importantly, the difference appears to be largely systematic, with dispersion energies calculated using monomer parameters being more binding than using parameters derived from the dimer wavefunction. Given we are fitting a model against the total energy, and the monomer wavefunction variant is significantly faster, we have decided to utilise this approximation by default, though the option to use the pair parameters is still implemented and included.

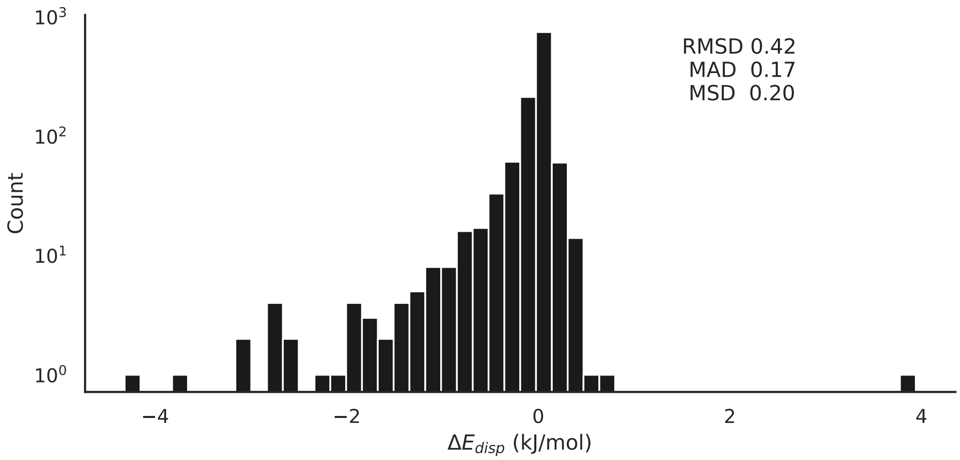

Fig. S1. Changes in XDM dispersion term for dimers used in the training dataset of this work, when using parameters from the monomer wavefunction vs. parameters from the orthogonalised dimer wavefunction, with the vertical axis being shown on a log-scale. The difference is largely systematic, and the correlation coefficient between the two is > 0.999. RMSD, MAD and MSD (all in kJ/mol) have also been provided in the figure.

S5. Fitting Procedure

Unless otherwise stated, all fits were performed using a least-squares procedure. Table S3 shows the variation between using the optimal (minimum RMSD) k scaling parameter for the CE-1p methods vs. using the global/transferable parameter.

Table S3. *Minimum fitted k values, and RMSDs for both the optimal k and $k = 0.78$, for all wavefunction sources examined in this work*

Method	Optimal k	RMSD (kJ/mol) k = Optimal	$k = 0.78$	Difference
HF	0.857	5.042	5.698	0.655
LDA	0.735	6.621	6.930	0.309
BLYP	0.694	6.777	7.533	0.755
B3LYP	0.735	4.954	5.239	0.285
ωB97X	0.776	4.222	4.267	0.045
ωB97M-V	0.776	4.299	4.344	0.045

Figure S2 shows the distribution of errors for choices of $k_{\text{exch-rep}}$ and k_{pol} parameters for the various wavefunction sources in this work.

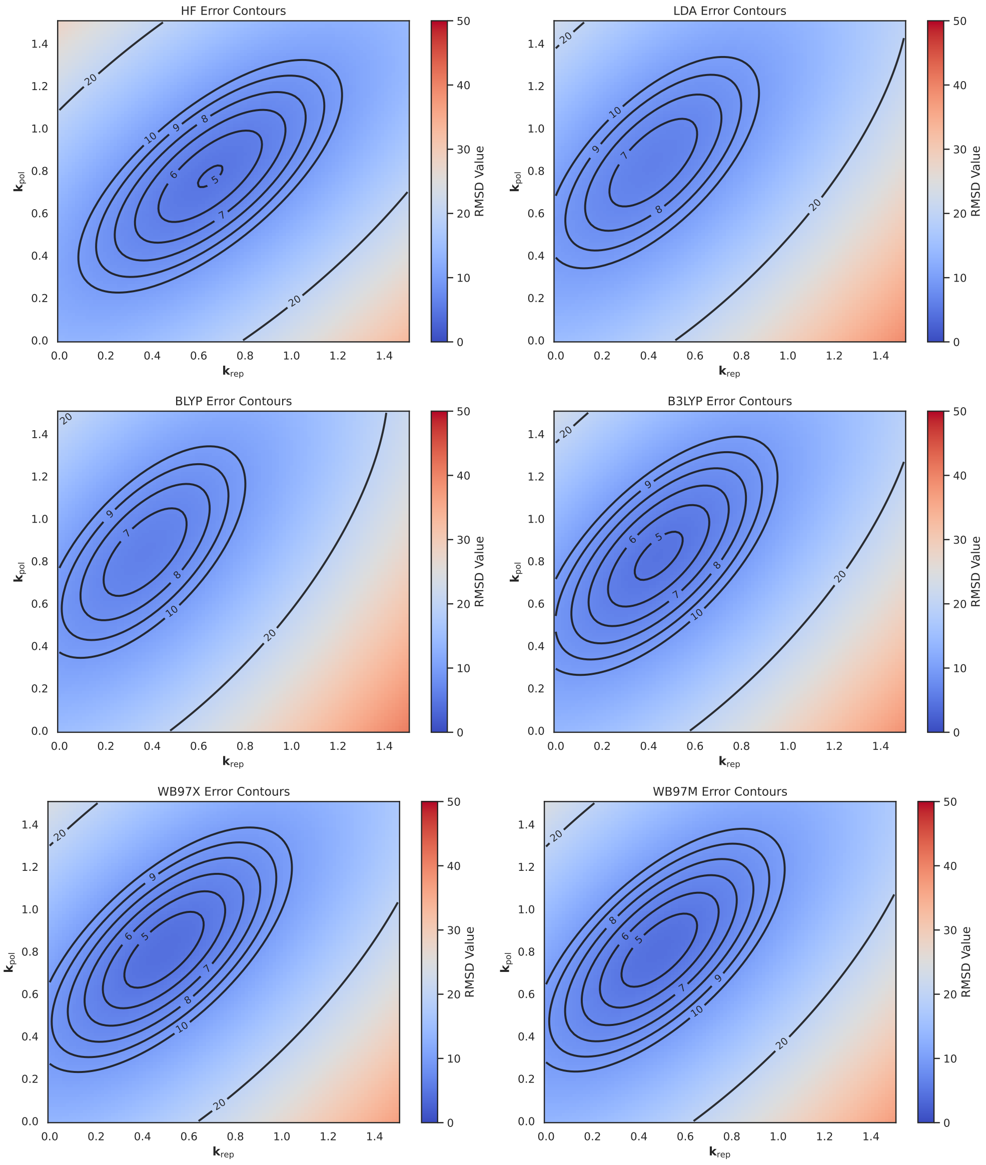

Fig. S2. Error contours for HF (top-left), LDA (top-right), BLYP (centre-left), B3LYP (centre-right), ωB97X (bottom-left) and ωB97M-V (bottom-right)

S6. S66x8

Table S4 shows errors for the S66 set for convenience and comparison to other works, itself a subset of the S66x8 set used throughout this work.

Table S4. *Mean Absolute Deviations (kJ/mol) for the S66 data set (i.e. the subset of the S66x8 where $r = 1.0$).*

method	model	MAD	σ
B3LYP	CE-B3LYP	2.52	2.64
	CE-1p	2.08	2.20
	CE-2p	1.86	1.46
	CE-5p	1.53	1.70
BLYP	CE-1p	2.80	3.55
	CE-2p	2.53	2.38
	CE-5p	2.06	2.03
HF	CE-1p	3.66	3.75
	CE-2p	4.23	5.17
	CE-5p	1.70	1.48
LDA	CE-1p	2.28	2.58
	CE-2p	1.91	2.08
	CE-5p	2.27	2.53
wB97X	CE-1p	1.78	1.90
	CE-2p	1.75	1.82
	CE-5p	1.55	1.72
wB97M-V	CE-1p	1.76	1.82
	CE-2p	1.58	1.70
	CE-5p	1.53	1.62

S7. Dimer interactions for high-pressure hydroquinone clathrates

Dimer interactions were calculated for all intermolecular interactions in the HQ-MeOH and HQ-MeCN clathrate crystals presented in (Eikeland *et al.*, 2017) in order to examine the behaviour of the newly fitted model when applied to crystals found under high pressure. Reference energies were calculated using the same reference as the training data i.e. ωB97M-V/def2-QZVP using ORCA, and compared to energies calculated using the new CE-1p model (with ωB97M-V/def2-svp for monomer wavefunctions), CE-B3LYP and GFN2-xTB.

Some results from these calculations are given Figure S3, where we can see that while the behaviour for CE-1p and CE-B3LYP is not the same at varying pressures, both exhibit significant increases in error as pressure gets higher (and thus interactions become closer). GFN2-xTB seems to be better behaved at these separations, likely due to it being fitted to reproduced forces and geometries.

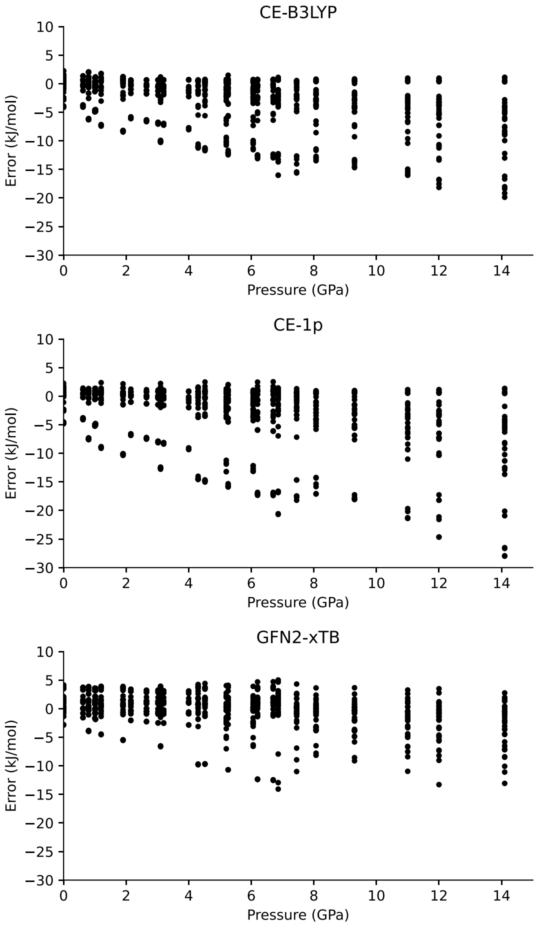

Fig. S3. Scatter plot of errors vs. ωB97M-V/def2-QZVP dimer energies for pairs in a set of hydroquinone clathrates with ethanol and acetonitrile. Errors are given in kJ/mol (y-axis) while pressures are provided in GPa (x-axis). Results are shown forCE-B3LYP (top), CE-1p using ωB97M-V/def2-svp (middle) and GFN2-xTB (bottom).

In order to probe the source(s) of the errors at closer separations, the error was plotted against the individual contributions E_{rep}, E_{ex}, E_{coul}, E_{disp} and E_{pol} to the total energy in the CE model, shown in Figure S4. Clearly there is the expected trend that a larger error is associated with larger energy terms, but there remains investigation to be done when examining the exact behaviour of e.g. the dispersion

interactions and the effects of damping factors which is outside the scope of this work.

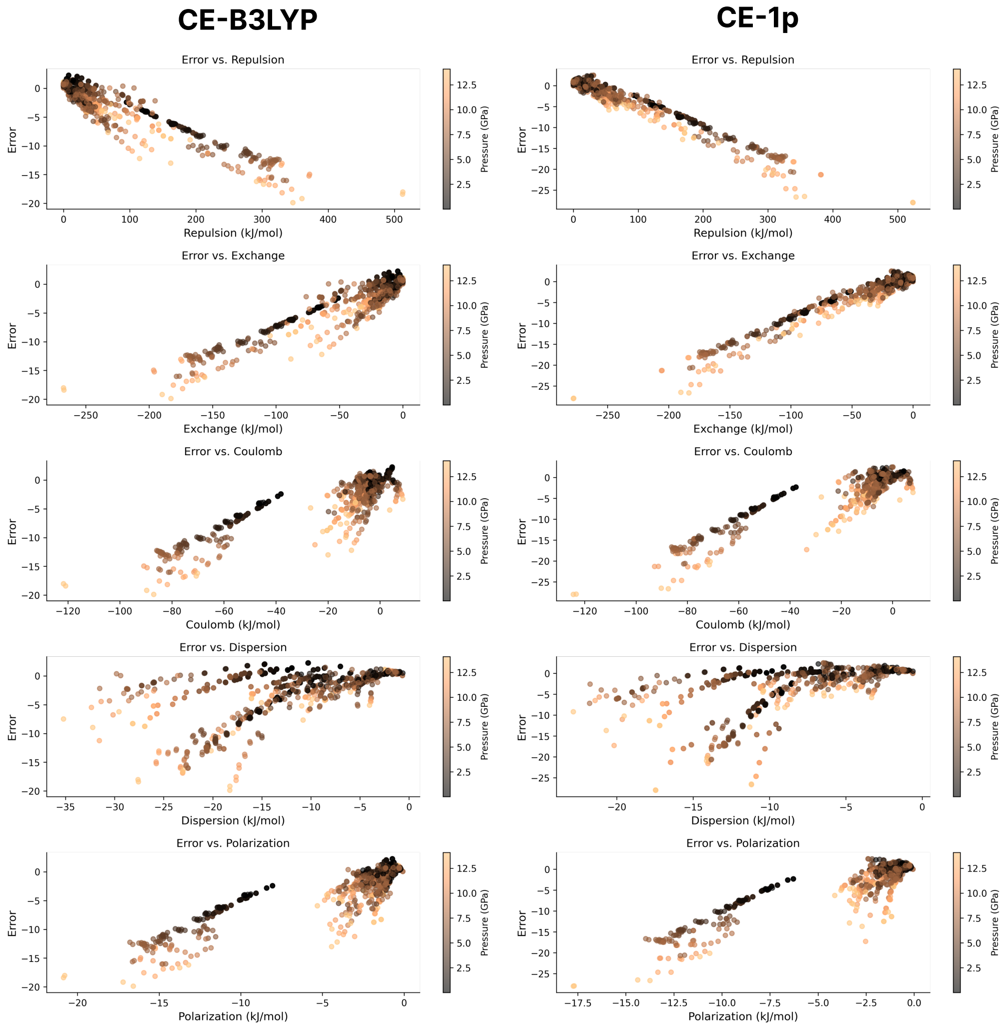

Fig. S4. Scatter plot of errors vs. ωB97M-V/def2-QZVP dimer energies for pairs in a set of hydroquinone clathrates with ethanol and acetonitrile. Errors are given in kJ/mol (y-axis) while pressures are provided in GPa (colour), and the energy components E_{rep}, E_{ex}, E_{coul}, E_{disp} and E_{pol} (top to bottom). Results are shown for CE-B3LYP (left)and CE-1p using ωB97M-V/def2-svp (right).

S8. X23 lattice energies

In previous work (Thomas *et al.*, 2018) there was a cell-dipole correction added to crystals in polar space groups, of a form shown below in Equation S7. This correction

has been omitted in this work, as we believe its inclusion to have been in error. The term itself is a correction added when using an Ewald summation, but since we are summing in molecular pairs out to very large cutoffs with vacuum boundary conditions no correction should be needed, see (van Eijck & Kroon, 1997). The net effect of its inclusion for the X23 set presented in this work is, in any case, minimal and the corresponding values have been provided in Table S5 for the four crystals in polar space groups.

$$E_{\text{cell-dipole}} = -\frac{2\pi p_{\text{cell}}^2}{3ZV_{\text{cell}}} \tag{S7}$$

Table S5. *Polar crystals in the X23 set, along with their corresponding CSD ref. codes, space group, crystallographic Z, cell dipole, p, and energy correction, E (see equation S7).*

Molecule	Ref code	Space group	Z	V_{cell} (Å^3)	p_{cell} (D)	$E_{\text{cell-dipole}}$ (kJ/mol)
cyclohexanedione	CYHEXO01	$P2_1$	2	290.08	2.237	-1.1
trioxane	TROXAN	$R3c$	6	638.28	15.098	-7.5
acetic acid	ACETAC01	$Pna2_1$	4	314.05	1.447	-0.2
pyrazole	PYRZOL02	$P2_1cn$	8	745.60	3.500	-0.3

A complete listing of the errors for the X23 benchmark set when using polarisation based on the crystal electric field is given in Table S6, while corresponding error values when using the experimental geometries (with X−H bonds normalised to values given in Table S2) is provided in Table S7.

Table S6. *Error Statistics for the X23 data set when incorporating the crystal-field polarisation corrections, showing Mean Absolute Deviation (MAD), Mean Signed Deviation (MSD) and Root-Mean-Square Deviation (RMSD) in kJ/mol for the CE-1p, CE-2p and CE-5p models investigated in this work.*

					CE-1p		
Statistic	CE-B3LYP	HF	LDA	BLYP	B3LYP	ωB97X	ωB97M-V
MAD	7.3	14.5	4.5	7.5	4.4	4.0	4.5
MSD	-6.3	-14.1	0.5	6.0	2.2	-0.7	-1.6
RMSD	9.7	18.5	5.7	8.4	5.3	5.2	5.6
					CE-2p		
Statistic		HF	LDA	BLYP	B3LYP	ωB97X	ωB97M-V
MAD		18.5	4.8	5.2	5.2	6.6	6.4
MSD		-18.3	-2.2	3.2	-1.4	-4.6	-4.6
RMSD		24.6	6.2	5.9	6.9	9.2	9.0
					CE-5p		
Statistic		HF	LDA	BLYP	B3LYP	ωB97X	ωB97M-V
MAD		6.3	9.1	10.4	7.0	5.1	5.3
MSD		4.6	-8.6	-9.8	-6.1	-3.8	-3.9
RMSD		8.8	11.1	12.7	9.1	7.1	7.2

Table S7. *Error Statistics for X23 set using experimental crystal structures with normalised hydrogen bond lengths, showing Mean Absolute Deviation (MAD), Mean Signed Deviation (MSD) and Root-Mean-Square Deviation (RMSD) in kJ/mol for the 1-parameter model investigated in this work.*

					CE-1p		
Statistic	CE-B3LYP	HF	LDA	BLYP	B3LYP	ωB97X	ωB97M-V
MAD	5.6	9.6	8.3	11.2	8.8	7.1	6.8
MSD	0.4	-6.2	6.9	10.9	7.7	5.3	4.8
RMSD	7.0	12.5	10.1	12.9	10.1	8.7	8.4
					CE-2p		
Statistic	GFN2-xTB	HF	LDA	BLYP	B3LYP	ωB97X	ωB97M-V
MAD	7.9	11.3	7.9	10.7	8.3	7.0	7.0
MSD	-0.7	-7.9	6.1	10.1	6.3	3.7	3.9
RMSD	10.3	15.3	9.6	12.1	9.7	8.8	8.6
					CE-5p		
Statistic		HF	LDA	BLYP	B3LYP	ωB97X	ωB97M-V
MAD		6.5	6.2	6.3	5.6	5.7	5.8
MSD		3.0	0.9	0.6	1.8	2.7	3.0
RMSD		7.7	7.9	7.9	7.3	7.5	7.5

S9. Repulsion term bound estimates

Both the exchange (E_{exch}) and repulsion (E_{rep}) terms are short-ranged, and so at certain intermolecular separations they can be ignored. However, using simple distance-based criteria for these bounds can be problematic as this overlooks the underlying

physics and the fact that the terms can decay at different rates for different systems. To alleviate this, we introduce a 'population difference' term based on the Mulliken population operator, defined as follows;

$$M = C_{AB}^{\mathrm{occ},\perp} - C_{AB}^{\mathrm{occ}} \tag{S8}$$

$$D^{\mathrm{diff}} = MM^T \tag{S9}$$

$$p = Tr[D_{AB}^{\mathrm{diff}}\ S_{AB}] \tag{S10}$$

where C^{occ} are the occupied orbitals, S_{AB} is the overlap matrix, $\perp$ indicates the orthonormalised pair wavefunction and D^{diff} is effectively a difference density matrix, p is then effective number of electrons which have shifted due to the orthonormalisation procedure. This metric gives a good linear correlation with the both E_{rep} and E_{exch} (correlation coefficients $r = 0.993$ and $r = 0.998$ respectively).

More importantly, p can be used to screen the calculation of the exchange and repulsion terms (and thus to introduce e.g. multipole-multipole interactions for the E_{coul} as well if desired) at very low cost, as seen in Figure S5. We would suggest a threshold of $p \leq 1 \times 10^{-6}$ as a conservative point to neglect the repulsion and exchange terms and introduce Coulomb approximations, which based on the data used in Figure S5 would result in a maximum error of 0.008 kJ/mol for repulsion and 0.004 kJ/mol for exchange, while eliminating 34/528 of the repulsion and exchange calculations. Likewise, a bound of $p \leq 1 \times 10^{-5}$ would eliminate 63/528 of the calculations, with maximum errors of 0.08 and 0.04 kJ/mol respectively, consistent with the linear relationship and roughly 1 order-of-magnitude differences. Such approximations would undoubtedly speed up e.g. lattice energy calculations with little to no change in the overall energy, particularly as the number of intermolecular interactions grows approximately as the cube of the distance, while this term rapidly decays with

distance.

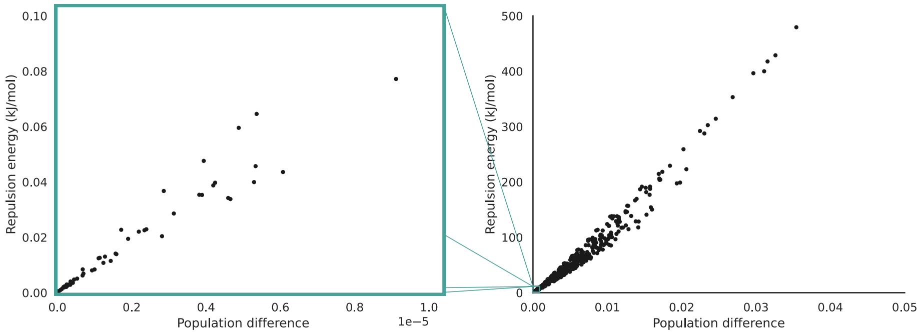

Fig. S5. Population difference vs. repulsion energy (see Equation S8 term for the S66x8 all dimers in the benchmark set, when using the ωB97M-V functional with def2-SVP. It should be noted that while the exchange term is not plotted here, the results are almost identical (with a sign difference, as E_{exch} is binding)

S10. References

References

van Eijck, B. P. & Kroon, J. (1997). *The Journal of Physical Chemistry B*, **101**(6), 1096–1100. **URL:** *https://doi.org/10.1021/jp962785u*

Eikeland, E., Thomsen, M. K., Overgaard, J., Spackman, M. A. & Iversen, B. B. (2017). *Crystal Growth & Design*, **17**(7), 3834–3846. **URL:** *https://doi.org/10.1021/acs.cgd.7b00408*

Thomas, S. P., Spackman, P. R., Jayatilaka, D. & Spackman, M. A. (2018). *Journal of Chemical Theory and Computation*, **14**(3), 1614–1623. PMID: 29406748. **URL:** *https://doi.org/10.1021/acs.jctc.7b01200*

iucr
